# Endoscopic Management of Primary Adenoid Cystic Carcinoma of the Nasal Septum: A Case Report and Literature Review

**DOI:** 10.7759/cureus.87509

**Published:** 2025-07-08

**Authors:** Takayuki Matsunaga, Shingo Umemoto, Takashi Hirano

**Affiliations:** 1 Otorhinolaryngology - Head and Neck Surgery, Faculty of Medicine, Oita University, Yufu, JPN

**Keywords:** adenoid cystic carcinoma, delayed metastasis, endoscopic surgery, nasal septum, postoperative radiotherapy

## Abstract

Adenoid cystic carcinoma (ACC) of the nasal septum is an exceedingly rare malignancy characterized by indolent but aggressive growth, frequent perineural invasion, and late-onset distant metastasis. Preoperative diagnosis can be challenging due to histopathologic similarities with benign salivary-type tumors. We report the case of a 75-year-old woman who presented with nasal obstruction and intermittent epistaxis. Nasal endoscopy identified a polypoid mass on the posterior nasal septum, and biopsy initially suggested a basal cell adenoma. Imaging revealed no evidence of perineural spread or skull base invasion. The patient underwent endoscopic resection with negative intraoperative margins. Final histopathological analysis confirmed cribriform-pattern ACC without perineural invasion. Given the patient’s comorbidities and family preference, adjuvant radiotherapy was not administered. Although the early postoperative course was uneventful, the patient developed local recurrence and cervical spine metastasis 52 months after surgery. She declined further treatment but remained alive with the disease at 72 months under observation. This case underscores the diagnostic complexity of nasal septum ACC and demonstrates the utility of endoscopic resection for localized tumors. However, in light of the delayed recurrence and close surgical margins typical of endoscopic approaches, the role of adjuvant radiotherapy should be carefully evaluated on an individual basis. Long-term follow-up is essential due to the potential for late recurrence and distant metastasis.

## Introduction

Adenoid cystic carcinoma (ACC) is a relatively rare malignancy of the head and neck region, most commonly arising from the salivary glands. Its occurrence in the nasal septum is exceedingly uncommon, with only a few cases reported in the literature published in English [1-5]. ACC is characterized by an indolent but aggressive clinical course, with a high propensity for perineural invasion and late-onset distant metastases [6]. Due to its rarity and histologic similarities with benign lesions, diagnosis is often delayed or initially inaccurate [3]. Here, we present a case of primary ACC of the nasal septum, successfully treated with endoscopic surgery, in which local recurrence developed during long-term follow-up. We then review the relevant literature to clarify the clinical features that support changes to the current management recommendations.

## Case presentation

History of present illness

**Figure 1:**
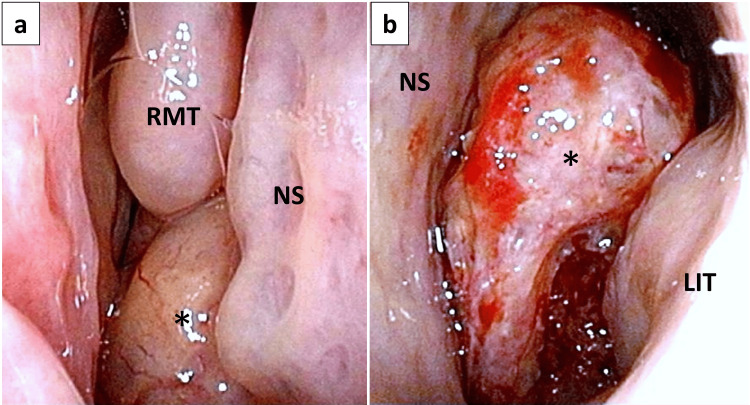
Nasal findings at initial examination. Right (a) and left (b) nasal endoscopic findings showing a hemorrhagic mass lesion that bled easily, originating in the left nasal septum and extending partway into the right nasal cavity, was found on examination of the left nasal cavity. The asterisks (*) indicate the tumor. NS: nasal septum; RMT: right middle turbinate; LIT: left inferior turbinate

A 75-year-old female patient presented with progressive nasal obstruction and intermittent epistaxis for one year. Nasal endoscopy revealed a vascular, polypoid mass on the posterior nasal septum (Figure 1). A punch biopsy suggested a basal cell adenoma (BCA). Although preoperative imaging raised some suspicion for malignancy, particularly due to bilateral extension through the nasal septum, the overall impression, based on the biopsy and the absence of definitive signs of invasion on imaging, was that of a benign lesion.

Therefore, we proceeded with endoscopic intranasal resection with curative intent. This approach was chosen due to the lesion’s location and the absence of skull base or orbital involvement, which made it technically feasible to achieve complete resection via a minimally invasive method. Before surgery, we informed the patient that although the biopsy was suggestive of a benign tumor, malignancy could not be completely ruled out, and that additional treatment such as radiotherapy might be necessary depending on the final pathology.

Past medical history and comorbidities

The patient had a history of left hemiplegia from a stroke 20 years earlier, chronic congestive heart failure, and persistent atrial fibrillation.

Imaging findings

**Figure 2:**
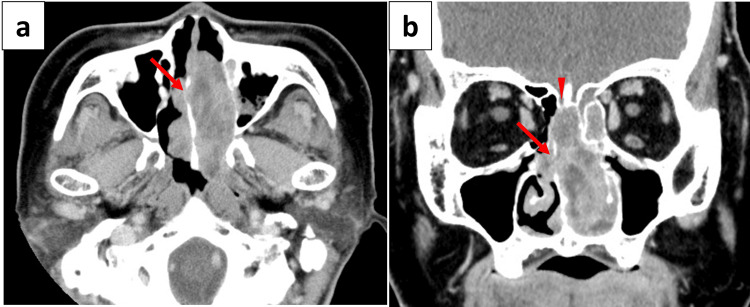
Preoperative sinonasal contrast CT findings. Axial (a) and coronal (b) sections showing the mass shadow with contrast effect centered on the nasal septum and extending into the left nasal cavity, left ethmoid sinus, and posterior nostrils, with no evidence of extension into the cranium, orbit, or hard palate, as interpreted by a board-certified radiologist at the time of initial evaluation. The arrows indicate the tumor. A retrospective review of the coronal image (b) reveals compression of the cribriform plate and subtle bone resorption, raising the possibility of involvement of the olfactory and ethmoid nerves (arrowhead).

Contrast-enhanced CT and MRI were performed to assess the tumor extent and involvement of adjacent structures. The CT revealed a mass shadow with contrast effect centered on the nasal septum and extending into the left nasal cavity, left ethmoid sinus, and posterior nasal aperture. Bony destruction of the nasal septum was also present (Figure 2).

**Figure 3:**
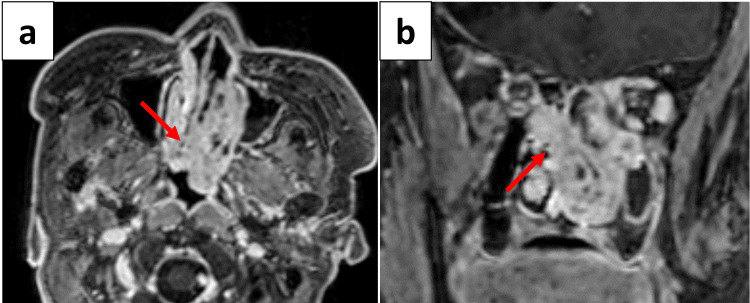
Preoperative sinonasal contrast MRI findings. Axial (a) and coronal (b) sections showing the mass signal with high contrast effect centered in the left nasal cavity. No obvious dural or orbital invasion was identified; however, the mass penetrated the nasal septum and extended into the right nasal cavity (arrows).

The MRI showed a contrast-enhancing mass signal centered in the left nasal cavity. No obvious perineural, dural, or orbital invasion was identified (Figure 3).

Surgical findings

**Figure 4:**
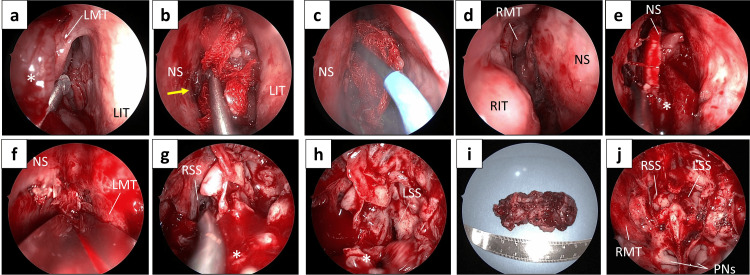
Operative findings. Multi-angle endoscopic views showing tumor localization, dissection, and complete en bloc resection with surrounding structures. (a) In the left nasal cavity, the tumor was partially adherent to the middle turbinate, but did not extend into the middle meatus. (b) The origin of the tumor was identified at the nasal septum (arrow). (c) An incision was made anterior to the tumor on the left side of the nasal septum. (d) The right side of the nasal septum was also incised to match the incision line on the left side. (e) The nasal septum and the left middle nasal turbinate were resected along with the tumor. (f) The nasal septum was resected along the line of the skull base along with the tumor. (g) The tumor did not extend into the right sphenoid sinus. (h) There was also no tumor extension into the left sphenoid sinus. The dorsal side of the tumor was dissected at the line of the anterior sphenoid wall, and the tumor was released. (i) The entire tumor was resected en bloc. (j) Nasal findings after tumor removal. The asterisks in panels a, e, g, and h indicate the tumor. LMT: left middle turbinate; LIT: left interior turbinate; NS: nasal septum; RMT: right middle turbinate; RIT: right interior turbinate; RSS: right sphenoid sinus; LSS: left sphenoid sinus; PNs: posterior nostrils

The patient underwent tumor resection under general anesthesia using an endoscopic intranasal approach. The tumor was partially adherent to the middle turbinate, although no extension into the middle meatus was observed (Figure 4a). The tumor base was identified on the left side of the nasal septum, where a mucosal incision was made (Figures 4b, 4c). A corresponding incision was also created on the right side of the septum (Figure 4d), and the tumor was resected en bloc along with the nasal septum (Figures 4e-4i). The left middle turbinate was resected in conjunction. No invasion posteriorly into the sphenoid sinus was identified on either side, and the tumor was transected at the anterior wall of the sphenoid sinus (Figure 4j). After complete release from the surrounding tissues, the tumor was removed in one piece. Intraoperative frozen sections confirmed negative surgical margins.

Postoperative course

**Figure 5:**
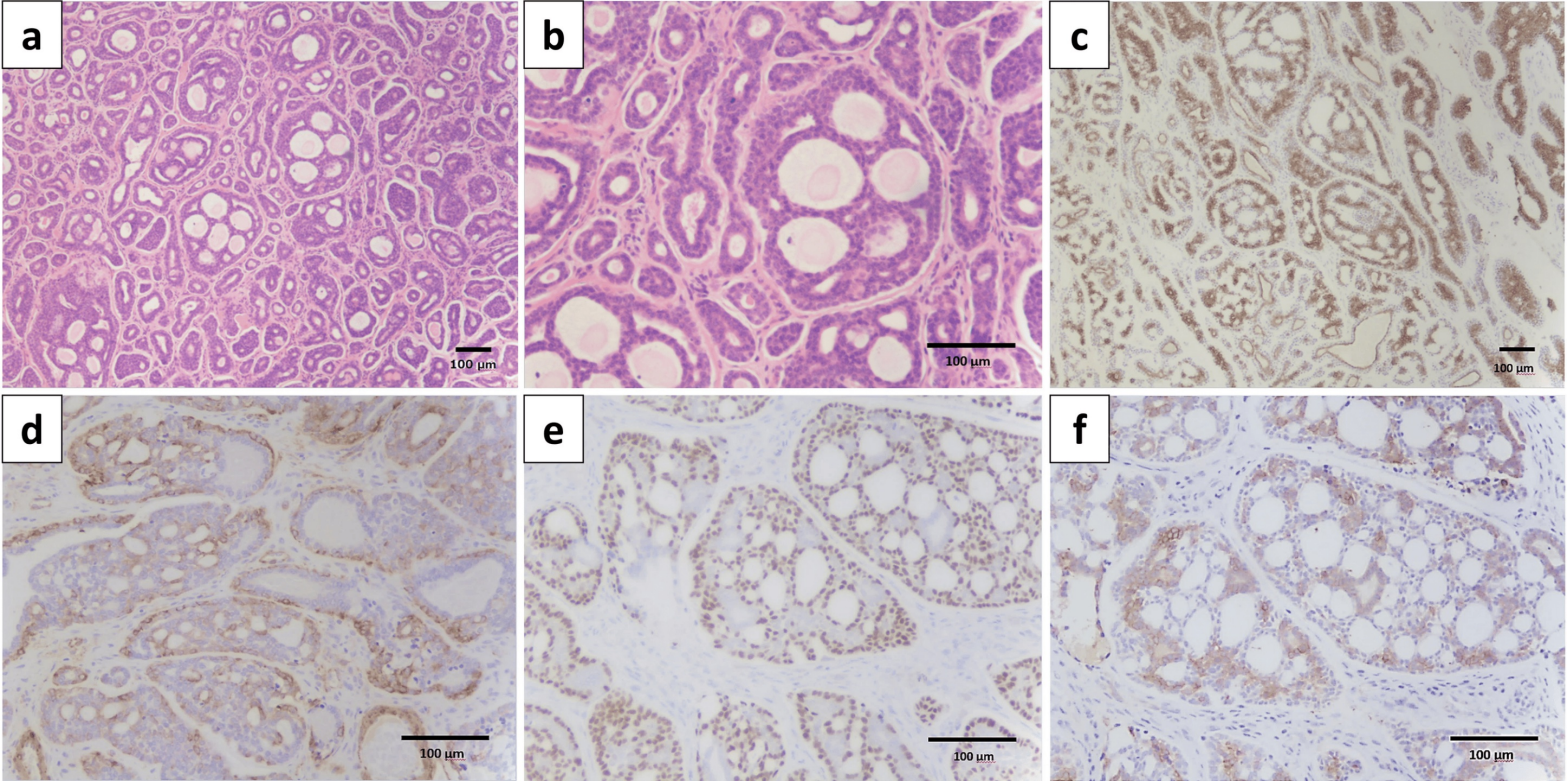
Histopathologic findings. The tissue consisted of two layers of conduit-like cells and myoepithelial/basal cell-like cells. The tumor cells showed an increase in nucleus-to-cytoplasm ratio with foci of tumor cells in a cribriform pattern. AE1/AE3, smooth muscle actins (SMA), p53, and c-kit immunostaining each showed positive results. Hematoxylin and eosin staining at low (a) and high (b) magnification; immunostaining for (c) pan-cytokeratin (AE1/AE3), (d) SMA, (e) p53, and (f) c-kit (CD117). Scale bars: 100 μm.

Histopathological examination of the excised specimen revealed a cribriform-pattern ACC. Immunohistochemical staining demonstrated positivity for pan-cytokeratin (AE1/AE3), smooth muscle actin (SMA), p53, and c-kit (CD117), supporting the diagnosis of ACC (Figure 5). Based on the postoperative histopathological and radiological findings, the tumor was classified as pT2N0M0, Stage II. Surgical margins were negative, and no evidence of perineural invasion was observed.

The postoperative course was uneventful. Although adjuvant radiotherapy was initially considered, it was ultimately withheld due to the patient’s comorbidities and the preferences of the patient and her family.

**Figure 6:**
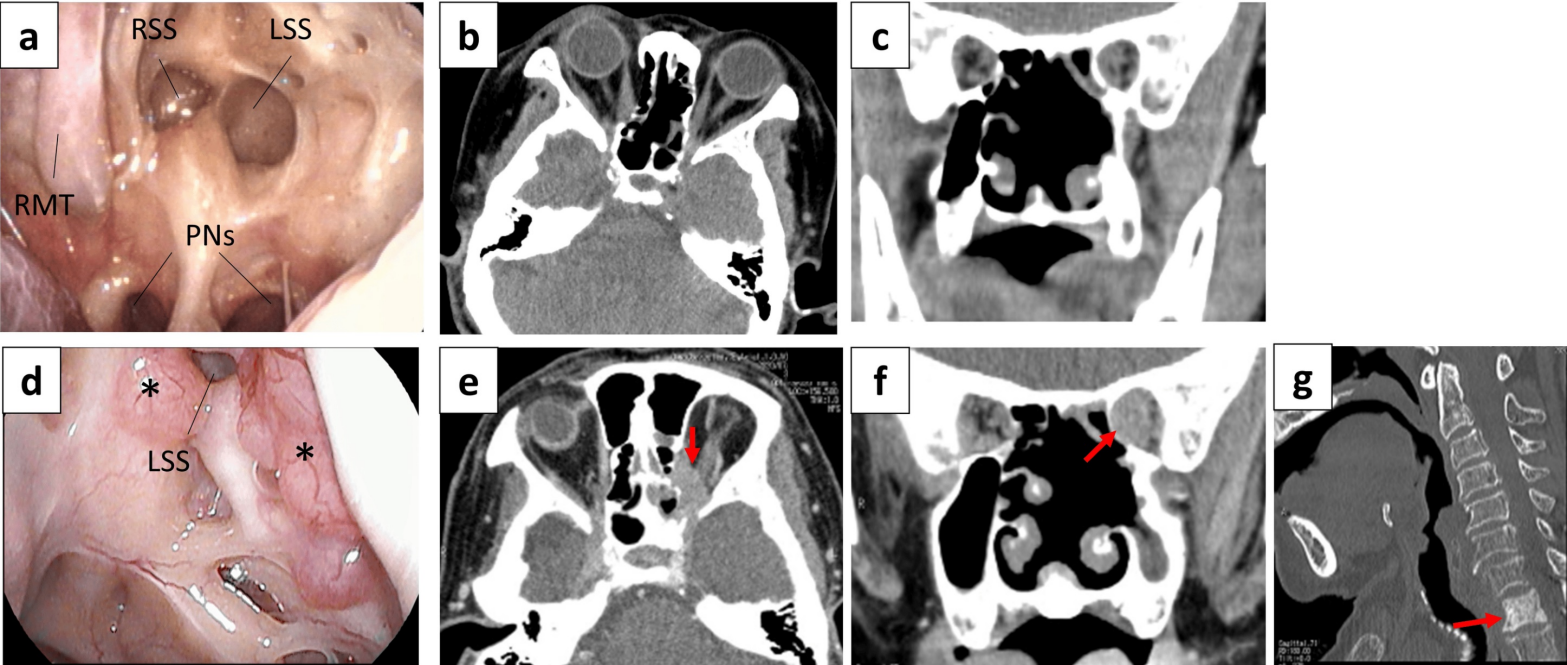
Postoperative clinical course. (a-c) Six-month follow-up with no tumor evident; (d-g) 52-month follow-up with recurrence of the nasal cavity tumor and metastasis to the 1st thoracic spine. At six months after surgery, there was no evidence of tumor in the nasal cavity (a). Axial (b) and coronal (c) CT sections did not reveal any tumor. However, at 52 months following surgery, a vascular, polypoid tumor was found in the nasal cavity (d), and axial (e), coronal (f), and sagittal spine (g). The asterisks (*) indicate the tumor. The arrows on panels e­­–g indicate recurrent tumor. RMT: right middle turbinate; RSS: right sphenoid sinus; LSS: left sphenoid sinus; PNs: posterior nostrils

The patient was followed closely with routine nasal endoscopy and imaging at regular intervals. At 52 months postoperatively, local recurrence was identified extending from the tip of the left orbit into the nasal cavity, accompanied by visual deterioration in the left eye. In addition, distant metastasis to the cervical spine was detected. However, in accordance with the patient’s wishes, no further treatment was administered. As of 72 months after surgery, the patient was alive with the disease and remains under clinical observation (Figure 6).

## Discussion

ACC is known for its indolent yet persistent growth, often leading to late-onset distant metastases and a decline in long-term survival. Whereas the five-year survival rate for patients with ACC is relatively favorable, ranging from 55% to 70%, the 10-year and 20-year survival rates drop substantially to 40% and 15%, respectively [7]. This steep decline underscores the indolent yet relentlessly progressive nature of ACC, which frequently manifests distant metastasis years after initial treatment. Given ACC’s propensity for late recurrence and metastasis, extended follow-up for at least 15 years is recommended [8].

Table 1 summarizes previously reported cases of ACC of the nasal septum, including the present case. Primary ACC of the nasal septum represents an exceptionally rare clinical entity within sinonasal malignancies. In previous reports [1-5], the predominant histological subtype in such cases has been cribriform. Typical presenting symptoms have included nasal obstruction and epistaxis [1-5], as in our case. Some patients have developed olfactory dysfunction due to perineural invasion by the tumor [4].

**Table 1:** Adenoid cystic carcinoma of the nasal septum case reports. M: male; F: female; N/A: not applicable

Author, year	Age (years), sex	Surgical procedure	Histological subtype	Postoperative radiation therapy	Postoperative recurrence	Observation period (months)
Schneiderman et al., 2002 [1]	66, M	Endoscopic intranasal resection	N/A	No	No	9
Sivaji et al., 2003 [2]	64, F	Lateral rhinotomy	Cribriform	Yes (planned)	No	N/A
Tai et al., 2007 [3]	56, M	Lateral rhinotomy	Cribriform	Yes (74 Gy)	No	12
Akiyama et al., 2013 [4]	42, F	Combination of anterior cranial, intranasal, and transpalatal surgery	Cribriform	Yes (50 Gy)	No	9
Kuruma et al., 2020 [5]	61, M	Endoscopic intranasal resection (four-hand operation)	Cribriform	No	No	36
Present case	75, F	Endoscopic intranasal resection	Cribriform	No	Yes (52 months)	72

In contradistinction to ours, most published reports describe outcomes over shorter follow-up periods, with none extending beyond five years (Table 1). The present case, featuring documented recurrence and metastasis at 52 months and survival at 72 months postoperatively, offers valuable insights into the long-term natural history of nasal septum ACC.

Diagnostic considerations

Accurate preoperative diagnosis of ACC can be challenging, particularly when evaluating limited or fragmented biopsy specimens. In our case, the initial biopsy suggested a benign BCA, highlighting the diagnostic complexity. This difficulty is well documented in the literature, where substantial histopathologic overlap between ACC and the other basaloid tumors, such as BCA, has been reported [9]. Both ACC and BCA share basaloid cytomorphology and can exhibit cribriform or tubular architectures, especially in small tissue samples [9].

Regardless of its architectural pattern, ACC is fundamentally a biphasic tumor composed of abluminal myoepithelial cells and luminal ductal cells [10]. In our case, immunohistochemistry demonstrated positivity for AE1/AE3, SMA, p53, and c-kit, a profile that is highly characteristic of ACC [9,11]. Numerous immunohistochemical studies have investigated the expression profiles of cytokeratins (e.g., AE1/AE3), S100 protein, SMA [10], epithelial membrane antigen, vimentin, and carcinoembryonic antigen. However, none of these markers has demonstrated sufficient specificity or consistency to be reliably used as a definitive diagnostic tool. Therefore, whereas immunohistochemistry serves as an important adjunct in the evaluation of ACC, diagnosis ultimately requires a comprehensive integration of histopathologic features and ancillary studies.

Among these immunohistochemical markers, c-kit expression was strongly and diffusely positive across the tumor cells in our patient, a finding strongly favoring ACC over BCA [11]. Whereas BCA may exhibit focal and weak c-kit positivity, the diffuse strong staining pattern observed in our case supports the diagnosis of ACC [11].

Thus, while the immunoprofile (AE1/AE3+, SMA+, p53+, c-kit+) was consistent with ACC, it is important to note that such a pattern could overlap with that of BCA, especially in small biopsy specimens. Consequently, immunohistochemical interpretation must consider not only the mere positivity or negativity but also the distribution, intensity, and pattern of staining across tumor components [9,11].

In summary, the combined histologic and immunohistochemical findings, including biphasic epithelial differentiation (AE1/AE3+, SMA+, p53+), strong diffuse c-kit positivity, presumed absence of nuclear β-catenin, and characteristic cribriform architecture, support the diagnosis of ACC. Therefore, this case underscores the difficulty inherent in making the preoperative diagnosis and the need to integrate morphologic, immunophenotypic, and molecular findings for accurate classification.

Imaging and surgical strategy

Radiological imaging is essential in both the preoperative assessment of ACC and surgical planning, as the images provide critical information regarding the extent of local invasion. ACC is well known for its propensity for perineural and hematogenous dissemination, rather than lymphatic spread [12], making high-resolution imaging particularly important for accurate staging and resection strategy. Contrast-enhanced CT and MRI serve complementary roles in the evaluation of sinonasal ACC. CT is superior in detecting cortical bone erosion and calcifications, whereas MRI excels in the soft tissue contrast resolution necessary for assessing perineural spread, periorbital extension, and potential skull base involvement. Several studies have emphasized the sensitivity of MRI for detecting perineural tumor extension, which is often underestimated clinically [13,14].

From a surgical standpoint, when the tumor is localized and technically resectable, endoscopic resection has emerged as an effective and minimally invasive approach. It offers superior intraoperative visualization and reduced morbidity, and facilitates en bloc resection with negative margins, particularly in early-stage or anatomically favorable tumors [1,15,16]. Recent advances in multi-hand, two-surgeon endoscopic techniques have further expanded the applicability of endoscopic surgery, reduced the need for external incisions, and enhanced functional and cosmetic outcomes in select patients [5].

Adjuvant therapy and prognosis

The role of adjuvant radiotherapy in ACC remains debated. Although the tumor is considered radiosensitive, it is not radiocurable. Radiotherapy is typically reserved for cases with positive or close margins, perineural invasion, or advanced disease. Notably, neutron radiotherapy has shown potential superiority over conventional radiation in select advanced cases, with improved local control reported in several series [17]. Garden et al. highlighted that patients with positive margins or perineural invasion achieved better local control with postoperative radiation therapy [18].

Thus, postoperative radiotherapy should be performed in advanced cases, of course, but also in early-stage cases, given that endoscopic intranasal surgery can only achieve a close margin.

Although surgery and radiotherapy remain the cornerstones of treatment for ACC, the role of chemotherapy remains controversial. Adjuvant chemotherapy is not routinely recommended due to the low mitotic activity and limited chemosensitivity of ACC. In recurrent or metastatic settings, systemic chemotherapy may be considered on a palliative basis, but its efficacy is limited. Current trials in targeted therapy and immunotherapy remain investigational, and no consensus has been established regarding their use in the adjuvant setting [19].

Distant metastases, often involving the lungs, bones, or brain, are not uncommon in ACC and can develop even in cases where local control has been achieved [20]. In addition, ACC is notorious for very late recurrence. Several studies have recommended that long-term follow-up extend beyond 15 years, as late recurrences, including distant metastases, can occur more than a decade after the initial diagnosis [8].

Reflections on treatment decision-making

Retrospective evaluation of the coronal CT image (Figure 2b) reveals compression of the cribriform plate and subtle bone resorption, which may suggest possible involvement of the olfactory and ethmoid nerves. Although these findings were not recognized at the time of preoperative imaging by a board-certified radiologist, they retrospectively indicate that a more aggressive or malignant lesion should have been considered.

Consequently, even though the tumor appeared macroscopically resectable with negative intraoperative margins, closer scrutiny of the preoperative imaging, particularly in the context of the cribriform architecture and vascular characteristics, might have raised stronger suspicion for malignancy. This could have prompted a more assertive preoperative diagnostic approach and earlier multidisciplinary discussion regarding adjuvant therapy.

Furthermore, while the decision to forgo postoperative radiotherapy was ultimately based on the patient’s and family’s wishes, we recognize that this choice may also reflect insufficient explanation by the clinical team regarding the importance of radiotherapy in the long-term control of ACC. Given ACC’s well-documented propensity for late recurrence, even in cases with negative margins and no perineural invasion, adjuvant radiotherapy should have been more strongly recommended.

In cases such as this, where complete oncologic clearance is difficult to achieve through endoscopic surgery alone, the omission of adjuvant radiotherapy may significantly compromise long-term disease control. We, therefore, reflect that, in this patient, adjuvant radiotherapy would likely have contributed to a reduced risk of recurrence and metastasis. This case serves as a valuable reminder of the critical importance of thorough perioperative counseling and shared decision-making, particularly in the management of rare and indolently progressive malignancies such as ACC.

## Conclusions

ACC arising in the nasal septum is a rare yet clinically important malignancy. Early detection, precise imaging, and complete surgical resection are central to favorable outcomes. Endoscopic surgery is a valuable approach for appropriately selected patients, especially those with localized disease. However, given the limited surgical margins typically achieved by intranasal approaches and the risk of late recurrence, adjuvant radiotherapy should be considered an essential component of treatment whenever feasible. In the present case, the omission of radiotherapy may have contributed to disease recurrence, highlighting the need for stronger preoperative suspicion, clearer communication regarding treatment options, and long-term clinical surveillance in patients with ACC.

## References

[1] Schneiderman TA, Chaudhury SI (2002). Adenoid cystic carcinoma of the nasal septum. Otolaryngol Head Neck Surg.

[2] Sivaji N, Basavaraj S, Stewart W, Dempster J (2003). Adenoid cystic carcinoma of the nasal septum. Rhinology.

[3] Tai SY, Chien CY, Tai CF, Kuo WR, Huang WT, Wang LF (2007). Nasal septum adenoid cystic carcinoma: a case report. Kaohsiung J Med Sci.

[4] Akiyama K, Karaki M, Hosikawa H, Mori N (2013). A massive adenoid cystic carcinoma of nasal septum progressed into the skull base. Auris Nasus Larynx.

[5] Kuruma T, Ogawa T, Arimoto M (2021). Endoscopic transnasal resection of adenoid cystic carcinoma of the nasal septum: a case report and literature review. Online J Otolaryngol Rhinol.

[6] Dantas AN, Morais EF, Macedo RA, Tinôco JM, Morais Mde L (2015). Clinicopathological characteristics and perineural invasion in adenoid cystic carcinoma: a systematic review. Braz J Otorhinolaryngol.

[7] Stawarz K, Durzynska M, Gałązka A (2025). Current landscape and future directions of therapeutic approaches for adenoid cystic carcinoma of the salivary glands (review). Oncol Lett.

[8] Castelnuovo P, Turri-Zanoni M (2020). Adenoid cystic carcinoma. Adv Otorhinolaryngol.

[9] Seethala RR (2017). Basaloid/blue salivary gland tumors. Mod Pathol.

[10] Speight PM, Barrett AW (2002). Salivary gland tumours. Oral Dis.

[11] Jung MJ, Roh JL, Choi SH, Nam SY, Kim SY, Lee SW, Cho KJ (2013). Basal cell adenocarcinoma of the salivary gland: a morphological and immunohistochemical comparison with basal cell adenoma with and without capsular invasion. Diagn Pathol.

[12] Chummun S, McLean NR, Kelly CG, Dawes PJ, Meikle D, Fellows S, Soames JV (2001). Adenoid cystic carcinoma of the head and neck. Br J Plast Surg.

[13] Wang Y, Guo X, Yu K, Shen X, Liu J, Zhao T, Gu H (2023). Adenoid cystic carcinoma of head and neck: summary and review of imaging findings. Heliyon.

[14] Sepúlveda I, Platin E, Delgado C, Rojas P (2015). Sinonasal adenoid cystic carcinoma with intracranial invasion and perineural spread: a case report and review of the literature. J Clin Imaging Sci.

[15] Kashiwazaki R, Turner MT, Geltzeiler M, Fernandez-Miranda JC, Gardner PA, Snyderman CH, Wang EW (2020). The endoscopic endonasal approach for sinonasal and nasopharyngeal adenoid cystic carcinoma. Laryngoscope.

[16] Volpi L, Bignami M, Lepera D (2019). Endoscopic endonasal resection of adenoid cystic carcinoma of the sinonasal tract and skull base. Laryngoscope.

[17] Mendenhall WM, Morris CG, Amdur RJ, Werning JW, Hinerman RW, Villaret DB (2004). Radiotherapy alone or combined with surgery for adenoid cystic carcinoma of the head and neck. Head Neck.

[18] Garden AS, Weber RS, Morrison WH, Ang KK, Peters LJ (1995). The influence of positive margins and nerve invasion in adenoid cystic carcinoma of the head and neck treated with surgery and radiation. Int J Radiat Oncol Biol Phys.

[19] Coca-Pelaz A, Rodrigo JP, Bradley PJ (2015). Adenoid cystic carcinoma of the head and neck--an update. Oral Oncol.

[20] Spiro RH (1997). Distant metastasis in adenoid cystic carcinoma of salivary origin. Am J Surg.

